# Malignant Syphilis in an Immunocompetent Patient: A Case Report and Review of the Literature

**DOI:** 10.3390/jcm14248839

**Published:** 2025-12-13

**Authors:** Chiara Vincenza Mazzola, Eleonora Bono, Ilenia Giacchino, Cinzia Calà, Luca Pipitò, Antonio Cascio

**Affiliations:** 1 Department of Health Promotion, Mother and Child Care, Internal Medicine, and Medical Specialties “G D’Alessandro”, University of Palermo, 90133 Palermo, Italy; chiaravincenza.mazzola@community.unipa.it (C.V.M.); eleonora.bono@community.unipa.it (E.B.); ilenia.giacchino@unipa.it (I.G.); cinzia.cala@unipa.it (C.C.); 2 Infectious and Tropical Diseases Unit and Sicilian Regional Reference Center for the Fight Against AIDS, AOU Policlinico “P. Giaccone”, 90133 Palermo, Italy; 3 Microbiology and Virology Unit, AOU Policlinico “P. Giaccone”, 90133 Palermo, Italy

**Keywords:** malignant syphilis, lues maligna, *Treponema pallidum*, immunocompetent, HIV-negative, secondary syphilis, cutaneous lesions, rupioid, molecular diagnosis, benzathine penicillin G

## Abstract

**Background**: Syphilis can present with diverse clinical manifestations, earning the name “great imitator.” Malignant syphilis (MS) is a rare, severe form of secondary syphilis, typically reported in immunocompromised patients, particularly those living with HIV. However, MS can occasionally occur in immunocompetent individuals, posing diagnostic challenges due to its atypical presentation. **Methods**: A case report is presented alongside a PubMed literature search using the terms “(malignant syphilis OR lues maligna) AND (immunocompetent) AND (case report OR case series).” No language or temporal restrictions were applied, yielding 18 relevant publications. **Results**: A 60-year-old HIV-negative man presented with fever, weight loss, papular lesions, and a single ulcer on the sternum. Serology was positive for syphilis, and PCR confirmed *T. pallidum* DNA in the lesion. Treatment with a single intramuscular dose of benzathine penicillin G led to prompt clinical and serological improvement. Literature review (*n* = 18) showed that MS in immunocompetent patients affects both sexes (55% male; mean age 37.1 years), often presents with ulceronodular or rupioid crusted lesions, and frequently involves systemic symptoms. Molecular diagnostics were rarely reported, with most diagnoses relying on histopathology and serology. Treatment with benzathine penicillin G was effective in all cases, and full recovery was achieved. **Conclusions**: MS can occur in immunocompetent, HIV-negative individuals without obvious risk factors. Clinicians should maintain a high index of suspicion in cases of systemic, cutaneous, or ocular manifestations suggestive of MS. Molecular assays can facilitate diagnosis and prevent unnecessary invasive procedures. Benzathine penicillin G remains the treatment of choice, demonstrating high therapeutic effectiveness. MS should be considered in the differential diagnosis of ulcerative or nodular dermatoses, regardless of immune status.

## 1. Introduction

Syphilis is a sexually transmitted infection caused by the spirochete *Treponema pallidum*, historically known as the “great imitator” due to its wide spectrum of clinical manifestations [1,2,3]. In 2023, the crude notification rate in 29 European Union and European Economic Area Member States was 9.9 cases per 100,000 population, a 100% increase since 2014, with the highest incidence among men aged 25–34 years and 72% of cases occurring in men who have sex with men (MSM), often associated with human immunodeficiency virus (HIV) infection [4].

After infection, syphilis typically progresses through primary, secondary, latent, and tertiary stages, ranging from painless chancre, regional lymphadenopathy, and polymorphic rash to gummatous, cardiovascular, or neurological involvement in the last stage [5]. In some cases, differential diagnosis is challenging and includes both other sexually transmitted infections and non-infectious conditions, such as Behçet’s disease, psoriasis, pityriasis rosea, and other conditions [5]. Malignant syphilis (MS) is a rare, severe form of secondary syphilis, usually seen in immunocompromised individuals, but it can occasionally occur in immunocompetent patients [6]. MS generally presents with prodromal systemic symptoms and a disseminated papulopustular rash that rapidly evolves into necrotic ulcers with rupioid crusts, sometimes accompanied by hepatic involvement with a notable increase in alkaline phosphatase level [6,7]. Diagnosis may be difficult due to atypical presentations and false-negative serology secondary to the prozone phenomenon [8]. Diagnosis is historically based on Fisher’s criteria, including high-titer serology, compatible morphology, a Jarisch-Herxheimer reaction (JHR) after treatment, and a dramatic response to antibiotics [9,10]. However, new diagnostic criteria have recently been proposed to include systemic manifestations, emphasizing musculoskeletal, central nervous system, ocular, ear, cardiovascular, rectal, liver, lung, and renal involvement [6]. While no specific regimen is formally established, a single intramuscular dose of 2.4 MU benzathine penicillin G (BPG) is generally sufficient [11,12].

MS remains a poorly understood condition, both in terms of its pathogenesis and its clinical manifestations. Although traditionally considered a rare complication of secondary syphilis and primarily associated with immunosuppression, malignant syphilis is increasingly recognized as an underdiagnosed entity in immunocompetent, HIV-negative individuals. Several factors contribute to this under-recognition, including atypical or misleading clinical presentations, which overlap with dermatologic or neoplastic conditions, and the limited awareness of malignant syphilis occurring outside HIV-infected populations.

Current diagnostic criteria have been developed largely from cohorts of patients living with HIV (PLWH), resulting in uncertainty about their applicability to immunocompetent hosts. In addition, the role of molecular testing remains poorly defined in this setting, despite its potential to provide rapid confirmation and avoid invasive procedures.

In this article, we describe a case of MS in an immunocompetent, HIV-negative patient whose initial presentation mimicked cutaneous lymphoma. Motivated by this case, we conducted a literature review. We aimed to delineate the epidemiological, clinical, and therapeutic characteristics of this population by summarizing all documented cases of MS in persons devoid of significant immunosuppressive factors.

Clarifying these aspects is highly relevant for contemporary clinical practice because clinicians increasingly encounter atypical dermatologic and systemic presentations of syphilis in both HIV-positive and HIV-negative populations.

## 2. Materials and Methods

We provide a detailed description of a case of MS in an immunocompetent patient without HIV infection. In addition to the case presented herein, we conducted a literature search on MS in immunocompetent individuals using only the PubMed database. We employed the search strings “(malignant syphilis OR lues maligna) AND (immunocompetent) AND (case report OR case series)”. No language or temporal restrictions were applied during the research. We yielded 18 relevant articles [13,14,15,16,17,18,19,20,21,22,23,24,25,26,27,28,29,30].

Studies were deemed eligible if they reported cases with a clinical diagnosis of malignant syphilis. Exclusion criteria included HIV infection and major immunosuppressive conditions, such as immunosuppressive therapy or solid-organ transplantation. Patients with common comorbidities potentially affecting immune function (e.g., diabetes mellitus, chronic kidney disease) were not excluded.

For each included case, we extracted demographic, clinical, diagnostic, and therapeutic data. We included both case reports and case series, for a total of 18 patients (see Table 1 [13,14,15,16,17,18,19,20,21,22,23,24,25,26,27,28,29,30]). Continuous variables were summarized as mean ± standard deviation or as median and interquartile range (IQR), whereas categorical variables were reported as absolute frequencies.

## 3. Case Presentation

**Figure 1:**
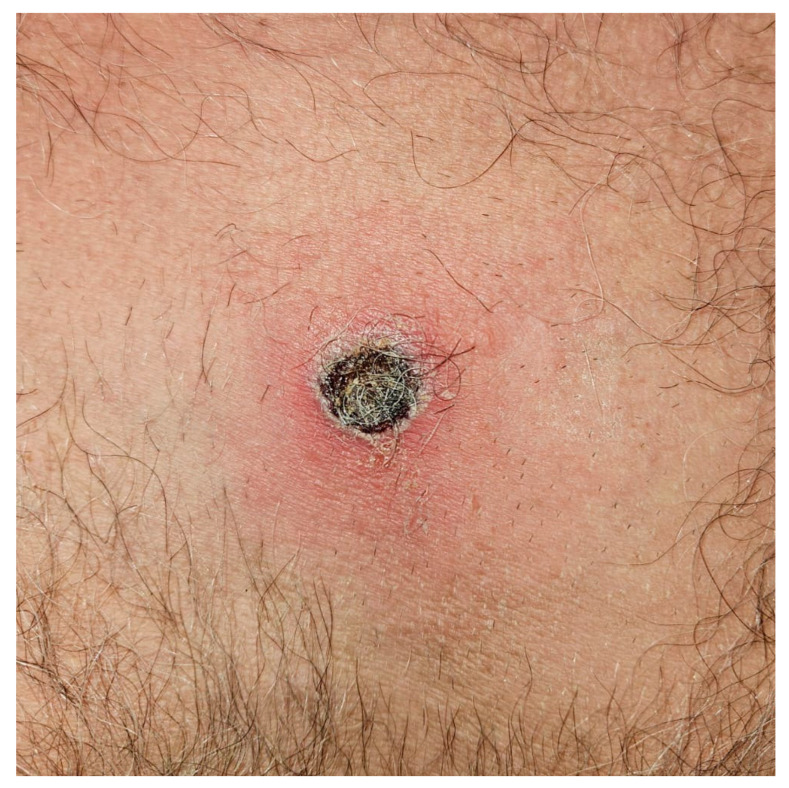
Ulcerated, solitary, painful lesion with raised edges, collarette scaling, and necrotic eschar (maximum diameter = 1.5 cm).

In 2024, a 60-year-old man was admitted to the Infectious and Tropical Diseases Unit with a three-month history of persistent fever (maximum temperature 38 °C), profuse sweating, progressive unintentional weight loss, and the development of multiple papular skin lesions, one of which had evolved into ulceration (Figure 1). He additionally reported paresthesia of the lower lip.

His past medical history included hypercholesterolemia (treated with rosuvastatin), inguinal hernia, and psoriasis, previously managed with biological therapy in 2021 and currently in washout. He was an active smoker.

At admission, vital signs were within normal limits except for fever and tachycardia (temperature 38 °C, blood pressure 125/80 mmHg, heart rate 117 bpm, respiratory rate 16/min, oxygen saturation 98% on room air). Physical examination revealed twelve papular lesions scattered across the trunk and back, and a single ulcerative lesion on the left parasternal area. The ulcer was covered by an eschar, surrounded by erythematous skin, and showed no exudate. Heart sounds were normal and rhythmic, with no murmurs. The patient exhibited poor oral hygiene with multiple carious teeth; no signs of meningeal irritation were noted. He reported a history of unprotected sex with male partners approximately five months before admission.

Baseline laboratory investigations showed C-reactive protein (CRP) 28.5 mg/L (normal < 5), aspartate aminotransferase 90 U/L (normal < 40), alanine aminotransferase 112 U/L (normal < 41), and alkaline phosphatase 338 U/L (normal 40–129). Serological testing for HIV, hepatitis B virus (HBV), and hepatitis C virus (HCV) was negative for active infection. Given the combination of systemic symptoms and an ulcerative skin lesion, cutaneous lymphoma was initially suspected, and a skin biopsy was planned. Furthermore, syphilis and tuberculosis were considered in the differential diagnosis. Treponemal serology tested positive, with a rapid plasma reagin (RPR) titer of 1:128 and total specific antibodies (chemiluminescent immunoassay, CLIA) > 70 index. Additionally, multiplex real-time Polymerase Chain Reaction (PCR, Genital Ulcer Assay [Allplex]), identifying herpes simplex virus type 1/2, *Haemophilus ducreyi*, cytomegalovirus, *Chlamydia trachomatis* serovars L1–L3, *Treponema pallidum*, and varicella zoster virus, was performed on the ulcer swab turned positive for *Treponema pallidum*, then skin biopsy was not performed. Comprehensive sexually transmitted infection screening, including *Neisseria gonorrhoeae*, *Chlamydia trachomatis*, *Trichomonas vaginalis*, and *Mycoplasma genitalium*, was subsequently performed on both first-void urine and rectal swabs by multiplex PCR, yielding negative results.

Therapy with ceftriaxone 2 g/day intravenously was initiated, preceded by 50 mg of prednisone to prevent a JHR, due to the unavailability of intravenous penicillin formulations. Ceftriaxone 2 g/day was administered for five days until the exclusion of central nervous system (CNS) involvement, with progressive reduction in CRP and disappearance of fever. To exclude CNS involvement, brain magnetic resonance imaging (MRI) and lumbar puncture were performed, both yielding normal results. Cerebrospinal fluid (CSF) analysis revealed no abnormalities, and CSF RPR was negative.

Subsequently, after ruling out neurosyphilis, a single intramuscular dose of 2.4 MU benzathine benzylpenicillin was administered. The patient showed rapid clinical improvement, with defervescence and normalization of liver enzymes and inflammatory markers.

At the six-month follow-up, the RPR titer decreased to 1:32, indicating a more than fourfold decline. The patient remained afebrile, asymptomatic, and free of relapse.

## 4. Review of the Literature and Discussion

### 4.1. Epidemiology and Medical History

All selected patients were tested for HIV, yielding seronegative results.

Of these, 55% were male, with ages ranging from 20 to 64 years (mean: 37.1 ± 11.8 years, median: 35 years [29–42.5]). The majority of patients did not present with significant comorbidities. A limited number of patients had mild comorbidities such as diabetes, chronic hepatitis, or thyroid disease [14,16,17,18,22], while only one had a previous history of monoclonal antibody therapy [18]. In our case, the patient’s medical history was notable for psoriasis previously treated with a monoclonal antibody, but the last administration occurred more than three years before presentation, so it cannot be considered a risk factor for MS presentation.

MS is most frequently observed in people living with HIV, including those with normal CD4^+^ T-cell counts under antiretroviral therapy, as well as individuals with uncontrolled HIV infection not receiving ART [7,31,32]. However, the pathogenesis of MS remains unclear. Other immunocompromising conditions have also been associated with MS, including diabetes mellitus, chronic kidney disease, malnutrition, and alcoholism [12]. Nevertheless, our observation supports the evidence that malignant syphilis can occasionally occur in immunocompetent hosts without evident risk factors, suggesting that other factors, such as transient immune dysregulation or prior immunomodulatory therapy, might play a contributory role in disease expression.

### 4.2. Clinical Features of Malignant Syphilis

Clinically, the majority presented with mixed ulceronodular lesions characterized by necrotic rupioid crusts, often involving the trunk and extremities. Our review showed that 16.7% were just ulcerated lesions [19,21,26], and 83.3% were both nodular and ulcerated [13,14,15,16,17,18,20,22,23,24,25,27,28,29,30]. Rupioid crusts were observed in 22.2% of cases [15,17,20,27]. Palmoplantar and mucosal involvement were documented in several cases, occasionally accompanied by genital or oropharyngeal lesions [13,14,16,17,18,26,27,28,30]. Systemic manifestations—including fever, malaise, lymphadenopathy, and weight loss—were frequently reported (83.3%). Of these, 55.5% presented with fever (T ≥ 38.5 °C) [13,14,15,16,17,18,19,21,22,23,24,25,26,28,29,30], 44.4% lymphadenopathy [13,15,16,17,21,26,28,30], 22.2% asthenia [20,26,27,30], 22.2% malaise [20,25,28,30], 22.2% weight loss [13,15,16,18], 16.6% myalgia [14,17,18], 16.6% anorexia [14,15,18], 11.1% low-grade fever (T < 38.5 °C) [20,27], and 11.1% arthralgia [24,28]. However, in 16.6% of cases, no constitutional symptoms were reported [19,22,29]. Clinical pictures included ocular involvement in two cases characterized by uveitis [21,22]. Laryngeal, pharyngeal [16], liver involvement [15], pulmonary manifestations [25], osteitis [25], and even facial nerve palsy [29] were also reported, highlighting the broad clinical spectrum of secondary syphilis. Our patient presented with systemic symptoms associated with a single crusted ulcer on the sternum and scattered papules on the trunk. An atypical manifestation was the referred lip paresthesia.

### 4.3. Diagnostic

Laboratory findings consistently demonstrated positive non-treponemal and Laboratory findings consistently showed positive non-treponemal and treponemal serology, with some reports describing the prozone phenomenon [17,30]. In our case, syphilis was diagnosed by serology and molecular analysis by PCR detecting *Treponema pallidum* DNA in the ulcer swab, confirming the etiological diagnosis and preventing unnecessary invasive investigations for differential diagnoses such as cutaneous lymphoma.

The application of molecular biology techniques, particularly PCR-based assays, has markedly enhanced the diagnosis of syphilis, especially in atypical or severe presentations such as malignant syphilis. In these complex cases, conventional serological tests may yield inconclusive results or be affected by the prozone phenomenon, leading to potential diagnostic delays [8,12,33]. Furthermore, negative results can be observed in immunocompromised patients, including those taking monoclonal antibodies [34].

Molecular methods enable direct detection of *Treponema pallidum* DNA in lesion swabs, tissue biopsies, or cerebrospinal fluid, allowing an earlier and more specific diagnosis. They are particularly useful in cases with atypical cutaneous manifestations, where they may help avoid more invasive diagnostic procedures [2]. Furthermore, molecular assays can support the differential diagnosis of malignant syphilis from other nodular and ulcerative skin conditions, such as disseminated fungal infections, cutaneous tuberculosis, vasculitis, and neoplastic lesions, where histopathological findings may be non-specific [7,31].

However, the use of PCR-based assays for the direct detection of *T. pallidum* DNA has important clinical and economic implications. In high-income settings, PCR is increasingly available in reference laboratories and can provide rapid, specific confirmation of syphilis in cases with atypical or severe cutaneous lesions. Although the cost of PCR exceeds that of serological testing, its application may be cost-effective in selected scenarios by reducing the need for invasive diagnostic procedures, avoiding misdiagnoses and preventing delays in initiating appropriate treatment. In resource-limited settings, PCR availability remains restricted. Our case illustrates how PCR confirmation can streamline diagnostic pathways, reduce healthcare costs associated with extensive investigations, and improve patient outcomes through earlier targeted therapy.

In the cases reviewed, no molecular assay was reported for lesion swabs, and biopsy was performed in all patients. Histopathology examination revealed dense plasma-cell–rich infiltrates, granulomatous dermatitis, and endarteritis obliterans. Spirochetes were detected by Warthin–Starry staining in 2 cases [24,27] and by immunostaining in 5 cases [15,18,19,25,27]. PCR testing for *T. pallidum* on a histological sample was reported in a case, with a positive result [16]. The results of blood examinations did not demonstrate any significant alterations. Indeed, an increase in WBC was observed in 5.5% of cases, elevated CRP in 22.2%, and elevated liver enzymes in 11.1%.

Lumbar puncture was performed in 27.8% of patients, with antibody positivity detected in a single case [16]. Also, in our case, we performed a lumbar puncture due to lip paresthesia, but both the lumbar puncture and CNS MRI excluded neurological involvement.

According to both the American CDC and IUSTI guidelines, lumbar puncture and CSF analysis are indicated in patients with syphilis who present with neurological, ocular, or otologic symptoms suggestive of neurosyphilis [35,36]. Routine CSF examination is not recommended in the absence of such findings. The CDC further notes that ocular or otosyphilis confirmed on examination should be treated as neurosyphilis regardless of CSF results [35]. In malignant syphilis, which represents a severe cutaneous manifestation of secondary syphilis, lumbar puncture is therefore not routinely required unless there are neurological or ophthalmologic signs suggestive of CNS involvement.

### 4.4. Treatment and Outcome

The majority of patients (83.3%) were treated with BPG, albeit with variation in dosage regimens: 66.6% received 2.4 MU/week intramuscularly for 3 weeks [13,14,17,18,20,21,22,23,24,26,27], 11.1% received 18 MU/day intravenously [15,16], and 5.5% received a once-only intramuscular injection of 2.4 MU [30]. A single patient received therapy with amoxicillin 2.25 mg/die in combination with probenecid [19]. Furthermore, penicillin allergy was documented in 11.1% of cases, thus necessitating the administration of doxycycline as an alternative treatment [25,28]. Given the unavailability of intravenous penicillin, our patient was initially treated with ceftriaxone for the first five days, until CNS involvement was excluded through lumbar puncture and brain MRI. Ceftriaxone has demonstrated non-inferiority to penicillin in a limited number of patients with neurosyphilis, and it is currently considered an acceptable second-line therapeutic option for this condition [37].

Subsequently, a single intramuscular dose of benzathine penicillin G led to gradual and complete resolution of both systemic symptoms and skin lesions, with a fourfold decrease in the RPR titer.

However, despite these cases reporting multiple administrations of BPG, current CDC and IUSTI guidelines suggest that malignant syphilis should be managed according to the treatment regimen for secondary syphilis, and no specific protocol is defined [35,36].

Moreover, to prevent the JHR, two patients received corticosteroids [16,17]. The JHR was described in 16.7% of cases and was generally mild [15,18,29]. In our case, we administered prednisone before starting treatment. However, corticosteroids are not routinely recommended but may be considered as adjunctive therapy in selected cases to prevent JHR, including malignant syphilis, ocular syphilis, and syphilis during pregnancy [12,35,38].

Finally, as in our case, full recovery was observed in all patients, and serological retesting data were reported in 38.9% of cases, with seronegativity occurring within 1 year of treatment [14,16,18,19,20,22,30].

## 5. Conclusions

Our review shows that MS can also occur in immunocompetent individuals without major immunodeficiency, presenting with clinical features comparable to those typically described in PLWH. Clinicians should therefore maintain a high index of suspicion when facing systemic, cutaneous, or ocular manifestations compatible with MS, even in the absence of known immunosuppression. A molecular assay to detect *Treponema pallidum* DNA can be considered to avoid unnecessary invasive investigations. BPG remains the treatment of choice and consistently demonstrates excellent efficacy across reported cases. In most cases, three doses of intramuscular BPG were reported, despite international guidelines suggesting a treatment according to secondary syphilis.

Finally, our findings emphasize the importance of including malignant syphilis in the differential diagnosis of ulcerative or nodular dermatoses.

The limitations of this review include the potential for publication bias. Malignant syphilis is not always readily identifiable, which may lead to misclassification. Additional limitations are the heterogeneity of the available reports and the lack of a structured assessment of their methodological quality.

## Figures and Tables

**Table 1:** Summary of published cases of malignant syphilis in immunocompetent individuals (2012–2025). Full recovery was observed for all the cases, but persistent scarring was reported in one case [19].

Author, Year	Sex/Age	Comorbidities	Lesion Type	Mucosal/Palmoplantar Involvement	Systemic Symptoms	Organ Involvement	Serology	Histopathology/PCR Findings	Therapy
Rao et al., 2017 [13]	M/35	None	Mixed	Yes/Yes	Fever, lymphadenopathy	Skin and Genitals	VDRL+	Endarteritis obliterans, plasma-cell infiltrate	IM BPG ×1
Sá Lopes et al., 2023 [14]	F/29	Hypothyroidism	Mixed	Yes/No	Fever, myalgia, anorexia	Skin	RPR+	Granulomatous dermatitis	IM BPG ×3
Xará et al., 2024 [15]	F/20	None	Mixed	No/No	Fever, anorexia, lymphadenopathy	Skin and Liver	RPR+	Granulomas, IHC+ for T. pallidum	IM BPG ×3
Dimnik et al., 2022 [16]	F/41	Chronic hepatitis	Mixed	No/Yes	Fever, lymphadenopathy	Skin, Laryngeal, nasal, gingival	RPR+	Granulomatous dermatitis, PCR+	IV penicillin G 14 days
Ortigosa et al., 2016 [17]	F/53	Diabetes	Mixed	Yes/No	Fever, myalgia	Skin	VDRL+FTA+	Interface dermatitis, granulomas	IM BPG ×3 + steroids
Requena et al., 2014 [18]	F/29	Psoriasis	Mixed	Yes/No	Fever, anorexia	Skin	VDRL+TPPA+	Lichenoid infiltrate, IHC+	IM BPG ×3
Barit et al., 2023 [19]	M/26	None	Ulcerative	No/No	None	Skin	RPR+TPPA+	Plasma-cell infiltrate, IHC+	Amoxicillin + probenecid
Alves et al., 2015 [20]	M/57	None	Mixed	No/No	Low-grade fever, asthenia	Skin	RPR+TPPA+	Granulomatous dermatitis	IM BPG ×3
Margulies et al., 2022 [21]	F/28	None	Ulcerative	No/Yes	Lymphadenopathy	Skin and Eyes (uveitis)	RPR+IgG+	Granulomatous lichenoid dermatitis	IV Penicillin G 14 days + steroid
de Unamuno Bustos et al., 2014 [22]	M/46	Hepatitis B	Mixed	No/Yes	None	Skin, genitals, and eyes (uveitis)	VDRL+FTA+	Inflammatory infiltrate, plasma cells	IM BPG ×3
García-Martínez et al., 2012 [23]	M/26	None	Mixed	No/No	Fever, malaise	Skin	VDRL+TPHA+	Lymphoplasmacytic infiltrate, endarteritis	IM BPG ×3
González-Lara et al., 2013 [24]	M/64	None	Mixed	No/No	Fever, arthralgia	Skin	RPR+	Plasma-cell infiltrate, WS+	IM BPG ×3 weeks
Rockwood & Nwokolo, 2018 [25]	M/41	None	Mixed	No/Yes	Malaise	Skin, genitals (testicular), lung, osseous	RPR+TPPA+	IHC+ for T. pallidum	Doxycycline 200 mg twice × 28 days
Demirbaş et al., 2022 [26]	M/35	None	Ulcerative	Yes/No	Asthenia, lymphadenopathy	Skin and genitals	RPR+TPHA+	Plasma-cell infiltrate	IM BPG ×3
Correia et al., 2022 [27]	M/30	None	Mixed	Yes/No	Fatigue	Skin	VDRL+TPPA+	Granulomas, WS+, IHC+	IM BPG ×3
Pradhan et al., 2018 [28]	F/35	None	Mixed	Yes/Yes	Fever, arthralgia	Skin and genitals	VDRL+TPHA+	Endarteritis obliterans	Doxycycline 100 mg twice ×21 days
Bertrand et al., 2012 [29]	F/43	None	Mixed	No/No	None	Skin and Facial nerve palsy (CNS)	RPR+	Plasma-cell infiltrate	IV penicillin G 14 days
Duenaz-Diaz et al., 2025 [30]	M/30	None	Mixed	Yes/No	Fever, asthenia	Skin	VDRL−(turned positive after 4 weeks), FTA+	Inflammatory infiltrate	IM BPG ×1

M—male; F—female; IM—intramuscular; IV—intravenous; BPG—Benzathine penicillin G; ×1—1 dose of 2.4 MU; ×3—3 doses of 2.4 MU once weekly for 3 weeks; WS—Warthin–Starry stain; IHC—immunohistochemistry; RPR—rapid plasma reagin; TPPA—*Treponema pallidum* particle agglutination assay; VDRL—Venereal Disease Research Laboratory; FTA—fluorescent treponemal antibody.

## Data Availability

The data presented in this study are available upon request from the corresponding author.

## Abbreviations

The following abbreviations are used in this manuscript:

CDC	Centers for disease control and prevention
CNS	Central nervous system
CRP	C-reactive protein
CSF	Cerebrospinal fluid
HBV	Hepatitis B virus
HCV	Hepatitis C virus
HIV	Human immunodeficiency virus
IUSTI	International Union against Sexually Transmitted Infections
JHR	Jarisch-Herxheimer reaction
MS	Malignant syphilis
MSM	Men who have sex with men
MRI	Magnetic resonance imaging
PCR	Polymerase chain reaction
RPR	rapid plasma reagin
VDRL	Venereal Diseases Research Laboratory
